# Smad signalling in the ovary

**DOI:** 10.1186/1477-7827-4-21

**Published:** 2006-04-12

**Authors:** Noora Kaivo-oja, Luke A Jeffery, Olli Ritvos, David G Mottershead

**Affiliations:** 1 Programme for Developmental and Reproductive Biology, Biomedicum Helsinki, University of Helsinki, Helsinki, Finland and Department of Bacteriology and Immunology, Haartman Institute, University of Helsinki, Helsinki, Finland

## Abstract

It has now been a decade since the first discovery of the intracellular Smad proteins, the downstream signalling molecules of one of the most important growth factor families in the animal kingdom, the transforming growth factor beta (TGF-beta) superfamily. In the ovary, several TGF-beta superfamily members are expressed by the oocyte, granulosa and thecal cells at different stages of folliculogenesis, and they signal mainly through two different Smad pathways in an autocrine/paracrine manner. Defects in the upstream signalling cascade molecules, the ligands and receptors, are known to have adverse effects on ovarian organogenesis and folliculogenesis, but the role of the individual Smad proteins in the proper function of the ovary is just beginning to be understood for example through the use of Smad knockout models. Although most of the different Smad knockouts are embryonic lethal, it is known, however, that in Smad1 and Smad5 knockout mice primordial germ cell development is impaired and that Smad3 deficient mice harbouring a deletion in exon 8 exhibit impaired folliculogenesis and reduced fertility. In this minireview we discuss the role of Smad structure and function in the ovarian context.

## Background

The main function of the female ovary is the production of the mature oocyte for fertilization to allow subsequent generation of healthy progeny. The ovary also has an endocrine function which is essential for the sexual maturation and reproductive ability of the female. The establishment of the germ line that gives rise to the oogonia and further the oocytes is therefore of fundamental importance for animal reproduction. Several growth factors belonging to the transforming growth factor β (TGF-β) superfamily together with their receptors and intracellular signalling molecules, the Smads, have been shown to be indispensable for the critical ovarian functions such as oocyte formation and development as well as ovarian folliculogenesis [1-3]. The temporal and spatial regulation of these signalling cascade molecules determines the responsiveness of the ovarian cell types to each stimulus during the course of folliculogenesis. Consequently, defects at either the growth factor, receptor or intracellular effector level may abrogate proper signalling and lead to adverse effects in fertility as is shown by extensive knockout studies in mice (for reference see [1]). In this minireview, we focus on the role of the different Smads, the downstream effectors of TGF-β superfamily ligands, in the ovarian organogenesis and folliculogenesis.

Proteins belonging to the Smad family were first identified in the fruit fly *Drosophila melanogaster *in the mid 1990s by Sekelsky et al. who found that an intracellular protein named Mad mediates the signalling of decapentaplegic (dpp), a member of the TGF-β superfamily corresponding to mammalian bone morphogenetic protein 2 or 4 (BMP-2/4) [4]. The discovery of orthologous proteins in *Caenorhabditis elegans *(Sma-proteins) as well as in vertebrates soon followed, and the newly found protein family was termed Smad [5]. Today, eight different members of the Smad family have been identified in mammals and an additional Smad4 identified in *Xenopus laevis *was termed Smad4β [6]. Based on their function, the Smads are classified as receptor-activated (R-) Smads (Smad1, -2, -3, -5 and -8), common-partner (Co-) Smads (Smad4, *Xenopus *Smad4α and Smad4β) or inhibitory (I-) Smads (Smad6 and -7). They function as intracellular transcription factors that mediate the signalling of the TGF-β superfamily which comprises now of more than 40 members including three isoforms of TGF-β, activins, inhibins, growth differentiation factors (GDFs) and bone morphogenetic proteins. All ligands in this protein family share common sequence elements and structural motifs. They are multifunctional regulators of cell proliferation, differentiation, migration and apoptosis, promoting extracellular matrix production, tissue homeostasis and embryogenesis. The TGFβ superfamily ligands are biologically active as homo- or heterodimers. As the ligand binds to the transmembrane type I and type II serine/threonine kinase receptors on the cell surface both receptor types dimerize forming a tetrameric signalling complex. Five type II Ser/Thr kinase receptors, and seven type I receptors have been identified so far. The type II receptor is considered to be constitutively active and it activates the type I receptor at its juxtamembrane GS-domain through phosphorylation. Furthermore, betaglycan and endoglin function as accessory receptors ("type III receptors") and modulate TGF-β activity. Depending on the combination of type I and type II receptors, different R-Smads are activated through phosphorylation by the type I receptor upon ligand binding. R-Smad2 and -3 are phosphorylated by activated TGF-β/activin type I receptors, and Smad1, -5 and -8 act downstream of BMP type I receptors. Activated R-Smads form oligomeric complexes with Co-Smad4 and translocate into the nucleus where they regulate target gene expression via interaction with a multitude of other transcription factors, co-activators and co-repressors. Inhibitory Smads block TGF-β superfamily signalling by binding to the type I receptors (Smad7) or by competing with activated R-Smad1 for binding to Co-Smad4 (Smad6). Smad7 can inhibit the activation of both the TGF-β/activin and BMP pathway R-Smads, whereas Smad6 is an inhibitor of BMP signalling [7-9].

Previously it was thought that the oocyte had a passive role in the events of folliculogenesis and the somatic granulosa cells were the main effectors. However, it is now well acknowledged that a bi-directional communication axis exists between the oocyte and its surrounding somatic cells; the oocyte regulates the differentiation and proliferation of the surrounding granulosa cells which in return secrete factors that modulate the growth of the oocyte itself [10,11]. Consequently, a complex interplay of regulatory factors governs the development of both cell types. In addition to pituitary endocrine hormones LH and FSH, a number of paracrine and autocrine factors, including several TGF-β superfamily members expressed by different ovarian cell types, contribute to the regulation of folliculogenesis. Gene expression studies have revealed in particular the BMP subfamily and their antagonists, as well as their transmembrane receptors and intracellular signalling molecules, play an important role throughout embryonic development and organogenesis. Concomitantly, these growth factors exhibit coordinated spatial and temporal expression patterns in fundamental cell types throughout the reproductive system and during gametogenesis. Gene knockout studies with mice have shown that certain BMPs and their downstream Smad effectors are required for germ line establishment. Both Smad1 and Smad5 knockout mice, although they die in the uterus during embryogenesis, show defective primordial germ cell (PGC) formation like BMP-4 and -8b deficient mouse embryos that survive gastrulation [3,12,13]. During ovarian folliculogenesis, Smad2 and Smad3 are expressed in a stage specific manner in the rat, possibly allowing different effects of the TGF-β superfamily ligands that use the same signalling pathways [14]. In the first part of this minireview we describe the structure and function of the Smad proteins as intracellular signalling molecules and present a multi-species alignment of the Smads that highlights the structural relatedness of the various Smad subgroups. In the second part we describe the Smad signalling pathways utilized by the different TGF-β superfamily ligands within the ovary and discuss the different ovarian phenotypes with defective Smad signalling.

### Smad structure

**Figure 1:**
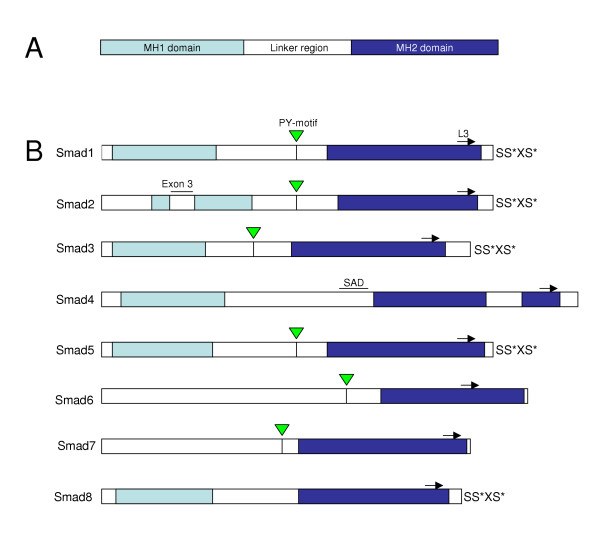
A schematic structure of the Smads. The N-terminal MH1 domain (light blue) and C-terminal MH2 domain (dark blue) are conserved among Smads. The non-conserved regions including the linker are shown in white. The two Serine (S) residues at the C-terminus of R-Smads that are phosphorylated by the type I receptor are marked with asterisks. MH Mad homology domain; PY, the proline-tyrosine (PPXY) motif identified by the E3 ligases Smurf1 and Smurf2; SAD, Smad4 activation domain.

The Smads contain two main domains that are conserved within the protein family, an N-terminal Mad homology 1 (MH1) domain and a MH2 domain in their C-terminus, that are connected by a non-conserved proline-rich linker region (Figure 1). The MH1 domain is highly conserved between the R-Smads and Co-Smads whereas the N-terminal region of the I-Smads shares only weak sequence similarity with the MH1 domain. In contrast, the MH2 domain is highly conserved among all Smads. The MH1 domain of the R-Smads and Co-Smads binds with low affinity to DNA recognizing specific sequences termed Smad binding elements (SBE) in the promoters of their target genes. However, the full length Smad2 lacks DNA-binding ability due to an insertion of the exon 3 coding for a 30-amino acid peptide in the MH1 domain [15]. Directed deletion of the exon 3 from full length Smad2 restores DNA binding ability allowing the expression of all essential downstream target genes of the TGF-β-related ligands [16]. The MH1 domain confers the ability to interact with other transcription factors and contains a nuclear localization sequence (NLS) and physically interacts with the MH2 domain exerting an autoinhibitory effect against Smad activation [17].

The linker region connecting the MH1 and MH2 domains is less conserved between the different Smads but contains several important regulatory peptide motifs, including potential phosphorylation sites for mitogen activated protein kinases (MAPKs). In addition, the linker region contains phosphorylation sites for the Erk-family MAP kinases [18], the Ca^2+^/calmodulin-dependent protein kinase II (CamKII) [19] and protein kinase C (PKC) [20]. A proline-tyrosine (PY) motif present in most R-Smads and I-Smads enables Smad interaction with the WW domains of the E3 ubiquitin ligases Smurf1 and Smurf2 (Smad ubiquitination-related factors) and SCF/Roc1 [21,22], and ubiquitin-mediated proteasomal degradation. Smad4 linker region also includes a nuclear export signal (NES) [23] and a Smad4 activation domain (SAD) that is required in transcriptional complexes mediating the activation of Smad-dependent target genes [24].

The MH2 domain is a multifunctional region that mediates type I receptor recognition through the L3 loop, transcriptional activation and R-Smad oligomerization with Smad4. It is also essential for cytoplasmic anchoring of the R-Smads as well as the interaction with Smad binding proteins and transcription factors. R-Smads are activated through phosphorylation of two serine residues at their C-terminal SSXS motif by the activated type I receptor at the inner leaflet of the cell membrane, whereas the I-Smads and the Co-Smads can not be phosphorylated C-terminally.

Analysis of the Smad protein sequences based on a combined cross-paralogue/cross-orthologue multiple sequence alignment (GoCore 5.0, Jeffery et al., manuscript in preparation; see references for the website address) highlights the structural relatedness of the various Smad subgroups (Additional file 1). The BRE pathway Smads (1/5/8) and the TGF-β/activin pathway Smads (2/3) form distinct groups. This grouping is particularly evident in the linker region, although this region otherwise exhibits a relatively low degree of homology across the R-Smad proteins. The grouping can also be observed in localized sites of the MH1 and MH2 domains.

### Smad signal transduction

Inactive R-Smads reside predominantly as monomers in the cytoplasm whereas the I-Smads localize to the cell nucleus [25,26]. Smad4 constitutively shuttles between cytoplasm and nucleus and its cytoplasmic localization in unstimulated cells is due to active nuclear export [23,27]. Cytoskeletal proteins also play a part in the localization and signalling of Smads. Unphosphorylated Smad2 and Smad3 bind microtubule filaments, and TGF-β treatment induces dissociation of these complexes [28]. Smads also interact with filamin, a scaffold for intracellular signalling proteins that crosslinks actin [29]. Upon ligand binding, the R-Smads are activated through C-terminal phosphorylation by the activated type I receptor at the inner leaflet of the cell membrane. A ligand can induce different signalling pathways depending on the composition of the receptor complex. Substrate specificity is determined by the L45 loop in the intracellular domain of the type I receptors and primarily by the L3 loop in the MH2 domain in the R-Smads [30,31]. Upon ligand binding, the activated type I and II receptors may be internalized via clathrin coated pits into early endosomes that contain a protein named SARA (Smad anchor for receptor activation) [32]. SARA is a FYVE domain containing scaffolding protein that interacts with the MH2 domain of inactive Smad2 and Smad3 targeting them to early endosomes and aiding in the recruitment of Smads to their receptors, thus promoting Smad phosphorylation and TGF-β signalling [33]. A recent discovery by Lin et al. reveals that a cytoplasmic protein named PML (promyelocytic leukaemia tumour suppressor) physically interacts with Smad2/Smad3 and SARA and is required for the association of Smad2 and -3 with SARA [34]. PML expression is induced by TGF-β and it is required for the accumulation of SARA and the TGF-β receptors in the early endosomes. Several other accessory/scaffolding proteins like SARA have been discovered for the TGF-β pathway R-Smads e.g. axin and disabled2 (Dab2) [35,36], but no accessory proteins have yet been discovered for the BMP-pathway Smads. Recently, Runyan et al. have shown that endocytosis of the receptor complex is required for proper nuclear translocation of activated Smads in human kidney mesangial cells, and that internalization enhances the dissociation of phosphorylated Smad2 from the TGF-β-receptor-SARA complex [37]. Internalization of the receptor complex through an alternate route, a lipid raft/caveolar dependent pathway, leads to the degradation of the receptor complex and thus regulates Smad activation and receptor turnover [32]. Following phosphorylation, Smads can form oligomers at different stochiometries; heterotrimers with two R-Smads and one Smad4 (Smad3) [38] or heterodimers consisting of an R-Smad (Smad2) and a Co-Smad [39]. Activated Smads accumulate into the nucleus where they control target gene expression in a cell type specific manner through interactions with other transcription factors, corepressors and coactivators. Diverse ligand responses in different cell types are a result of different Smad-interacting transcription factors and of cooperation with other signalling pathways.

### Modulation of Smad activation

TGF-β superfamily signalling is modulated at multiple levels. Extracellular ligand trapping molecules or antagonists, including gremlin, noggin, chordin (all members of DAN/Cerberus protein family) and follistatin, can block ligand binding to the receptors. Another antagonist is the naturally occurring pseudoreceptor BAMBI (BMP and Activin membrane bound inhibitor) which extracellularily resembles a type I receptor but lacks the cytosolic kinase domain. BAMBI can form stable associations with various TGF-β family type I receptors thus blocking BMP, activin and TGF-β signalling [40]. Receptor internalization provides another point of modulation of the signal transduction pathway as mentioned above. Also within the cell, alterations in Smad protein levels can profoundly affect signalling. The basal levels of Smad proteins are regulated post-translationally through a ubiquitin-mediated proteasomal degradation pathway [41,42]. Both the size of the Smad pool in unstimulated cells and the levels of activated Smads are thus regulated. The E3 ligases, Smurf1 and -2, as well as SCF/Roc1, antagonize TGF-β family signalling through interaction with R-Smads, targeting them for degradation and terminating Smad-mediated signalling [22,41,43]. Therefore, Smurf-mediated degradation regulates R-Smad levels and the sensitivity of cells to incoming signals.

In contrast to R-Smad expression, expression of the inhibitory Smad6 or Smad7 is regulated by extracellular signals. Induction of Smad6 and Smad7 expression by BMP and TGF-β, respectively, represents an auto-inhibitory feedback mechanism for ligand-induced signalling [8,44]. Recruitment of a complex of Smad7 with either Smurf1 or Smurf2 to the type I TGF-β receptors at the cell membrane results in receptor ubiquitination and degradation. Internalization of the receptor complex bound to Smad7 via lipid raft caveolin-positive compartments promotes poly-ubiquitination and results in accelerated receptor turnover [32]. Activation of the epidermal growth factor (EGF) receptor, and possibly other tyrosine kinase receptors, interferon-γ signalling through STAT (signal transducer and activator of transcription) proteins, and activation of NF-κB by tumour-necrosis factor-α, also induce Smad7 expression [45]. In addition to the Smurfs, inhibitory Smad6 and Smad7 also modulate the Smad-mediated signalling. Smad7 inhibits both TGF-β and BMP pathway Smad activation through interaction with the type I receptor, whereas Smad6 blocks only BMP pathway Smads by competing with the activated R-Smads for heteromeric complex formation with Co-Smad4 [25]. Co-repressors c-Ski and SnoN are two highly conserved members of the Ski family of proto-oncoproteins and they can antagonize TGF-β signalling through direct interactions with Smad4 and the R-Smads inhibiting transcription of target genes [46].

Members of the MAP kinase family are frequently involved in TGF-β/Smad signalling and nuclear accumulation of activated Smads can be modulated by Ras activated Erk kinases. Epidermal growth factor (EGF), hepatocyte growth factor and oncogenic Ras stimulate Erk kinases, which in turn phosphorylate Smad proteins. Erk phosphorylates serine residues in the linker regions of Smad1 [18], Smad2 and Smad3 [47]. Phosphorylation of Smads can also result from the activation of MAPK/Erk kinase kinase 1 (MEKK1), which acts downstream from Ras and upstream from the growth factor-induced Erk MAPK and stress-activated SAPK/JNK pathways [48]. Phosphorylation of Smads in the MAP kinase sites at the linker region attenuates ligand-induced nuclear translocation and alters Smad-dependent transcription. Dephosphorylation of the Smads by as yet unidentified phosphatases is another mechanism for the termination of Smad signalling. Activation of Ca2+/calmodulin-dependent protein kinase II (CamKII) also results in Smad2, Smad3 and Smad4 phosphorylation inhibiting TGF-β-induced nuclear import and transcriptional activity of Smad2, and affecting Smad heteromerization [19]. Protein kinase C (PKC) activity abrogates DNA binding of Smad3 [20]. Also Smad7 can be phosphorylated independently of TGF-β stimulation at Ser249 [49], whereas it is unknown whether Smad6 is modulated by phosphorylation. Smad7 phosphorylation does not affect TGF-β signalling but rather the TGF-β independent effect of Smad7 on transcriptional regulation [49]. In conclusion, phosphorylation of the Smads not only causes their activation but also modulates their activity and provides a mechanism for integration of the Smad pathway with other signalling pathways that can modulate TGF-β superfamily signalling.

### TGF-β superfamily ligands in ovarian organogenesis and folliculogenesis

The establishment of the germ line is of fundamental importance to animal reproduction. In mice, the extraembryonic ectoderm induces germ cell determination [50]. During early embryonic development in mice, uncommitted epiblast cells in the extra-embryonic mesoderm involute through the primitive streak to the yolk sac endoderm and become committed as primordial germ cells (PGCs). These cells proliferate and migrate via the yolk sac into the hind gut endoderm and dorsal mesentery, and finally to the genital ridges during gastrulation. Upon reaching their destination, PGCs lose their motility, become encapsulated by the primary sex cords and differentiate depending on the sex chromosome set up into oogonia or spermatogonia. The cortical sex cords give rise to the female ovaries, whereas the medulla slowly deteriorates [51]. The process of gametogenesis starts as the PGCs leave the dorsal mesentery and continues as they enter and colonize the genital ridges to establish the prospective gonad. The role of the TGF-β superfamily ligands in ovarian organogenesis as well as folliculogenesis has been studied extensively in animals. In particular, the BMPs together with their antagonists have been shown to be prominent throughout embryonic development and organogenesis. Gene ablation studies in mice have identified BMP4, -8b and -2 as regulators of primordial germ cell (PGC) formation from epiblast cells, BMP-2 deriving from the embryonic endoderm and BMP-4 and -8b from the extra-embryonal ectoderm [3]. Targeted mutations of either BMP-4 or BMP-8b lead to severe defects in PGC formation in the embryos that survive gastrulation [3]. Also, altered germ cell migration in the absence of TGF-β signalling via ALK-5 has been reported [52].

Within the established ovary, the progress of folliculogenesis is in part regulated by peripheral endocrine factors, the pituitary gonadotropins FSH and LH as well as growth hormone (GH) and prolactin in some species. In addition, intraovarian factors, such as steroids, cytokines and other growth factors act in a paracrine/autocrine manner and co-ordinately contribute to the processes of recruitment, development, atresia, selection and ovulation of follicles [53]. The growth of the follicle is considered gonadotropin-independent to the small antral stage and during these early phases folliculogenesis appears to be driven by the local autocrine and paracrine signals from the oocyte and the surrounding somatic cells. A complex bi-directional communication between the oocyte and granulosa cells as well as between the granulosa and thecal cells drives the progression of follicular development through successive stages [54]. Various TGF-β superfamily ligands expressed by the different ovarian cell types are important in this interaction and their expression is regulated in a developmental-stage related manner. Among the local factors at least activins, inhibins, TGF-β s, BMP-6, GDF-9 and its homologue GDF-9B (also known as BMP-15) as well as anti-Müllerian hormone (AMH, also known as Müllerian inhibiting substance, MIS) are implicated in having a role during the development of follicles (for review, see [51]). The developing oocyte has been shown to express GDF-9, GDF-9B, BMP-6 and TGF-β 2, although the TGF-β protein may not be secreted [55-59]. Granulosa cells produce activins, inhibins, TGF-β s, BMP-2, BMP-3 and BMP-6 as well as AMH at different stages of folliculogenesis, while the theca cells have been reported to produce all the isoforms of TGF-β, BMP-3b, BMP-4 and BMP-7 [60,61]. TGF-β s, activins and GDF-9 signal through the Smad2/3 pathway whereas GDF-9B, BMP-2, -4, -6 and -7 utilize the Smad1/5/8 pathway (see Table 1). Table 1 summarizes the different superfamily ligands expressed by the oocyte, granulosa cells and thecal cells as well as their receptors and the Smad pathways they activate when known.

**Table 1:** Signalling pathways of TGF-β superfamily ligands expressed in the ovary.

**Ligand**	**Expressed by**	**Type II receptor**	**Type I receptor**	**Smads**	**References**
GDF-9	oocyte	BMPRII	ALK5	Smad2/3	[86-88]
GDF-9B/BMP-15	oocyte	BMPRII	ALK6	Smad1/5/8	[91]
BMP-6	oocyte, granulosa cell	BMPRII/ActRIIA/B	ALK2/ALK3/ALK6	Smad1/5/8	[94-96]
TGF-β 1, -2, -3	granulosa and theca cell	TβRII	ALK5	Smad2/3	[132, 133]
Activin A/B	granulosa cell	ActRIIB	ALK4	Smad2/3	[134, 135]
Inhibin α	granulosa cell	ActRIIA/ActRIIB	?	?	[136]
BMP-2	granulosa cell	BMPRII/ActRIIA	ALK3/ALK6	Smad1/5/8	[137, 138]
BMP-3	granulosa cell	ActRIIB	?	?	[106]
AMH/MIS	granulosa cell	AMHRII	ALK2/ALK3/ALK6	Smad1/5/8	[111, 113-115]
BMP-3b	theca cell	?	?	?	[139]
BMP-4	theca cell	BMPRII/ActRIIA	ALK3/ALK6	Smad1/5/8	[137, 138, 140]
BMP-7	theca cell	BMPRII/ActRIIA	ALK2/ALK3/ALK6	Smad1/5/8	[140, 141]

For appropriate signalling, an intact signalling cascade from ligands and receptors to intracellular effectors and accessory proteins has to be present and functional. The temporal and spatial regulation of these signalling cascade molecules determines the responsiveness of the cell type to each stimulus, and the direction of signalling within the follicle is dependent on cellular distribution of the whole signalling pathway. Reproductive defects can be found in knockout mice at all levels of the signalling cascade; at the ligand level activin β B, inhibin α, GDF-9, GDF-9B and AMH are known to cause fertility defects, at the receptor level, ALK6 (also known as BMP type IB receptor) and AMH type II receptor (AMHRII) affect female fertility, and finally at the intracellular effector level, Smad3 knockout mice exhibit reduced fertility [1,2,62]. Ingman et al. very recently reported that TGF-β 1 null mice show severely impaired reproductive capacity and almost complete infertility [63]. In the following section we discuss the main TGF-β superfamily ligands expressed by the different follicle cell types; the oocyte, granulosa and thecal cells as well as their target cells and signalling pathways.

#### The oocyte

In most mammalian species, both GDF-9 and GDF-9B mRNA and protein expression begin at the primary follicle stage and continue throughout the development of the maturing follicle [55,56,64]. Depending on the species, both GDF-9 and GDF-9B are indispensable for normal progression of ovarian folliculogenesis as shown by the mouse and sheep animal models [65-67]. GDF-9 deficient mice display arrested follicular development at the primary follicle stage, the theca cell layer is absent and in addition their oocyte development is compromised. Therefore, the homozygous female mice are infertile while heterozygous females and male mice are not affected [65]. A naturally occurring mutation in the GDF-9 gene has been discovered recently in sheep that causes sterility in homozygous ewes due to abnormal follicle development, but surprisingly, increased ovulation rate and fertility in heterozygotes [67].

In contrast to the mouse GDF-9 knockout, female mice completely lacking GDF-9B are fertile but exhibit reduced fertility due to defects in the ovulation process and the ability of oocytes to develop into normal embryos, whereas heterozygous females exhibit normal fertility [68]. In sheep, four different mutations in the GDF-9B gene have been identified that affect fertility and ovulation rate, introducing premature stop codons (Belclare and Cambridge sheep FecX^G ^or Hanna mutation FecX^H^) or non-conservative amino acid substitutions within the mature protein (Inverdale FecX^I ^or Belclare FecX^B^) (reviewed in [69]). All these mutations cause an arrest in folliculogenesis at the primary follicle stage in homozygous animals similar to the phenotype of the GDF-9 knockout mouse. In contrast, heterozygous ewes exhibit increased ovulation rates and fertility. The reason for these difference in phenotypes between mice and sheep is not fully understood but it has been suggested, however, that the differences may derive from the different ovulatory nature of these species, the sheep being a low ovulation rate species and the mice a poly-ovulatory species, or the different relative importance of these growth factors in sheep and mice [68,70].

The biological functions of the oocyte secreted GDF-9 have been studied extensively over the past five years, and an essential role for GDF-9 in the early stages of folliculogenesis as well as during ovulation is emerging. Recombinant GDF-9 functions as a granulosa cell mitogen and has been shown to modulate granulosa cell steroidogenesis. In addition, it has been shown to stimulate the growth of preantral rat follicles and the proliferation of rat and mouse granulosa cells [71-74] as well as to induce the cumulus cell phenotype during ovulation [72]. Only a few target genes for GDF-9 have been identified so far. GDF-9 has been shown to induce granulosa cell hyalurononan synthase 2 (Has2), cyclo-oxygenase 2 (Cox2), and steroidogenic acute regulatory protein (StAR) mRNA expression and to suppress the protease urokinase plasminogen activator (uPa) and luteinizing hormone receptor (LHR) as detected with semi-quantitative RT-PCR [72,75]. With a microarray approach, Varani et al. found that GDF-9 induces pentraxin 3 expression in mural granulosa cells from preovulatory mouse follicles [76] and recently, Pangas et al. identified gremlin, a BMP antagonist, as a gene regulated by GDF-9 in mouse granulosa cells from large antral follicles [77]. GDF-9 may also modulate theca cell function as it is known to stimulate the expression of CYP17, a theca cell marker [78], and androgen biosynthesis in rat theca-interstitial cells [79] as well as to inhibit 3'5'-adenosine monophosphate-stimulated steroidogenesis in human theca cells [80].

Few targets for GDF-9B have been identified, however, it is known to suppress FSH receptor mRNA expression [81], to stimulate Kit ligand expression in rat granulosa cells [82] and to simultaneously promote expression of anti-apoptotic Bcl-2 and suppress pro-apoptotic Bax [83]. Recombinant GDF-9B functions as a granulosa and cumulus cell growth factor by actively preventing cell death and promoting DNA synthesis and proliferation *in vitro *[81,83]. In the light of recent data it is becoming clear that GDF-9 and GDF-9B can co-operate to regulate granulosa cell functions e.g. proliferation and gonadotropin-induced differentiation [84,85].

GDF-9 has been shown to mediate its signal through cell surface receptors BMPRII, that normally functions as a BMP type II receptor, and ALK5, the type I receptor of TGF-β, and activates Smad2/3 pathway [86-90]. The GDF-9 receptor combination is interesting since this is the first reported physical interaction between ALK5 and a BMP type II receptor. GDF-9B interacts with BMPRII and has also been shown to interact with ALK6 (or BMP receptor type IB) and causes the activation of Smad1/5/8 pathway [91]. The receptor complex binding a GDF-9-GDF-9B heterodimer has not been reported yet, but it could be predicted to consist of two BMPRII molecules in complex with one ALK5 and one ALK6 molecule. Interestingly, a naturally occurring point mutation was found in sheep in the gene coding for ALK6 (Booroola gene FecB), the type I receptor for e.g. GDF-9B, which causes increased ovulation rates in heterozygous sheep compared to wild type sheep, and even higher ovulation rates in homozygotes [92]. Although GDF-9B, BMP-2, -4, -6 and -7 are expressed in the mammalian ovary and can signal through ALK6, it is not known to what degree each of these is involved in the Booroola phenotype. However, based on the similarity of the phenotype with the heterozygous Inverdale ewe with a point mutation in the GDF-9B protein coding gene, the involvement of altered BMP-15 signalling is strongly suspected [92], GDF-9B showing the highest affinity to ALK6 of the type I receptors [91]. The follicles of these Booroola ewes mature and ovulate at smaller sizes with fewer granulosa cells than in wild-type ewes. The ALK6 knockout mouse phenotype differs from the Booroola sheep phenotype. The knockout mice appear to have normal ovarian follicular development and ovulation rates, but display reduced fertility which may be caused by the failure of normal cumulus expansion [2].

BMP-6 is the third TGF-β superfamily growth factor secreted by the oocyte from the primary stage onwards [58] but it lacks the mitogenic activity of GDF-9 and GDF-9B (Gilchrist et al., 2005 submitted) [93]. BMP-6 modulates granulosa cell steroidogenesis by inhibiting FSH-induced progesterone synthesis, but has no effect on estradiol production. BMP-6 suppresses the FSH action at the level of adenylate cyclase downstream of the FSH receptor in contrast to GDF-9B which suppresses FSH receptor expression [81,93]. The preference of cell surface receptors for BMP-6 in the ovary has not been determined yet but BMPRII, ActRII as well as ActRIIB have been implicated as type II receptors for BMP-6 [94,95] and all BMP ALKs (ALK2, -3 and -6) have been identified as potential BMP-6 type I receptors, with ALK6 having the strongest binding affinity [95,96]. BMP-6 can activate the Smad1/5/8 pathway in a human granulosa tumour cell line [91].

#### The granulosa cells

Activins and inhibins were first discovered as gonadal proteins that regulate pituitary FSH secretion [97]. Three types of activin are produced in the ovary by the granulosa cells, each consisting of a dimer of two related subunits β A and β B i.e. homodimeric activin A (β Aβ A) and activin B (β Bβ B), and a heterodimeric activin AB (β Aβ B). Two types of inhibin are also expressed by granulosa cells. Inhibins consist of one inhibin α-subunit and one activin subunit forming either inhibin A (α-β A) or inhibin B (α-β B). Activin produced by the secretory gonadotrophs in the anterior pituitary stimulates FSH production in a paracrine manner, and within the ovary activin promotes granulosa cell proliferation [98] as well as potentiates FSH actions by increasing FSH receptor expression [99]. Activin also modulates granulosa and theca cell steroidogenesis. Activins are produced by the granulosa cells and the expression pattern of the different activin subunit mRNAs changes during folliculogenesis.

Inhibins are also produced by the granulosa cells, and they act as endocrine hormones that are released into the circulation to suppress pituitary FSH production. Locally, inhibins also act as potent regulators of activin signalling. Inhibins compete with activin signalling by blocking activin binding to type II activin receptors. β-glycan, an inhibin co-receptor, facilitates inhibin binding to the activin type II receptor [100]. Follistatin (FS) is yet another granulosa cell produced inhibitor of activin function which also regulates the actions several BMPs, including GDF-9B/BMP-15 [83]. Follistatin antagonises activin through forming biologically inactive complexes. Activin subunits bind ActRIIB and ALK4, and activate the Smad2/3 pathway whereas the inhibin α-subunit binds to ActRIIA or ActRIIB. Cripto, a prototypic member of the epidermal growth factor-Cripto protein family, antagonises activin signalling by binding to the activin type II receptor and blocking ALK4 recruitment [101].

Also BMP-2, BMP-3 and BMP-6 are expressed by the granulosa cells [102,103]. Recombinant BMP-2 has been shown to amplify FSH-induced estradiol and inhibin A production in sheep granulosa cells [104] and stimulate inhibin β B subunit mRNA expression as well as inhibin B protein production in cultured human granulosa luteal cells [105]. BMP-2 can signal through either BMPRII or ActRIIA and ALK3 and -6 activating the Smad1/5/8 pathway. BMP-3 mRNA has been shown to be expressed in human granulosa luteal cells but the biological function of BMP-3 in the ovary is still unclear. However, it was reported that the expression level of BMP-3 is regulated by human chorionic gonadotropin (hCG) [103]. Recently, it was discovered that BMP-3 binds ActRIIB and functions as a novel inhibitor for both activin and BMP-4 signalling in *Xenopus *embryos [106]. BMP-6 expression in granulosa cells is first detected at the early secondary stage in rat follicles and it is rapidly lost at the time of dominant follicle selection suggesting that inhibition of BMP-6 gene activity may be required for the formation of the dominant follicle. BMP-6 is again highly expressed in atretic follicles supporting this hypothesis [102].

AMH is expressed by the Sertoli cells in the testis and granulosa cells in the ovary [107]. In the male AMH causes the regression of Müllerian ducts that in the female differentiate into the oviducts, the uterus and the upper part of the vagina. In the granulosa cells AMH is first expressed postnatally in primordial follicles recruited to growth and continues to be expressed until the growing follicles are selected for dominance by the action of FSH. AMH deficient mice are fertile but their pool of primordial follicles is depleted earlier than wild type mice [108]. AMH has been shown to inhibit the initiation of primordial follicle growth in the neonatal mouse ovaries as well as inhibit the stimulatory effect of FSH on the growth of preantral and small antral follicles [107]. Instead, in the rat, AMH promotes preantral follicle growth in the presence of FSH but not preantral follicle cell differentiation and apoptosis [109] and in human, it was recently found that AMH induces growth of primordial follicles from ovarian cortical tissue [110]. AMH binds to the AMH type II receptor [111] and causes the activation of Smad1/5/8 in a granulosa tumour cell line [112], but the type I receptor has yet to be conclusively confirmed (ALK2,-3 and -6 are implicated) [113-115].

#### The theca cells

A theca cell layer forms to surround the developing follicle outside the basal lamina at the primary/secondary transition. Theca cells from rat follicles have been shown to produce BMP-3b, BMP-4 and BMP-7, as well as all the isoforms of TGF-β [102,116,117]. Recombinant BMP-4 and -7 have been found to modulate FSH signalling by promoting FSH induced estradiol production and inhibiting progesterone biosynthesis [116]. Granulosa cells in growing follicles produce estradiol but no progesterone *in vivo *in response to FSH stimulation until the periovulatory period, whereas *in vitro *cultured granulosa cells produce progesterone as well as estradiol in response to FSH stimulation. Therefore, it has been suggested that the biological function of theca cell derived BMPs might be to function as selective inhibitors of progesterone synthesis (luteinization inhibitors) as neither BMP-4 or BMP-7 affect granulosa cell steroidogenesis in the absence of FSH in the rat [116]. Both BMP-4 and -7 bind to the BMPRII/ActRIIA receptors and ALK3 and -6 which are predominantly expressed in granulosa cells [116], BMP-7 may also signal through ALK2 [118]. Both activate the Smad1/5/8 pathway in their target cells.

In conclusion, the oocyte secreted factors GDF-9, GDF-9B and BMP-6 can activate either Smad2/3 or Smad1/5/8 pathways in the granulosa cells and modulate their differentiation and proliferation. However, Smad2/3 signalling is by far the predominate pathway used by oocyte secreted factors to promote granulosa and cumulus cell growth and cumulus cell expansion (Gilchrist et al., submitted). It is not clear, however, to what extent Smad mediated signalling is involved in the oocyte maturation process during folliculogenesis or whether the oocyte secreted factors have autocrine effects on the oocyte itself. The human oocyte shows immunostaining for Smad2 and Smad4 at primordial and primary stages of folliculogenesis, having therefore the capacity to respond to TGF-β-like ligands but little is known of the significance of the Smad1/5/8 pathway in the oocyte development [119]. The granulosa cell expressed ligands may act locally in a paracrine or autocrine fashion affecting the granulosa cells themselves or the thecal cells and possibly even the oocyte, or they may have systemic effects acting on for example the pituitary gonadotropin expression of activins and inhibins.

### What do transgenic mouse models tell about Smad signalling in the ovary?

The downstream effectors of the TGF-β superfamily ligands, the Smads, are expressed ubiquitously throughout development in practically all adult tissues and are also essential for normal embryonic development. Although the targeted disruption of individual receptor-regulated Smad genes in mice (Smad1, -2, -4, or -5) results in the death of homozygous embryos, the knockout models have nevertheless revealed different roles for the Smads at different stages of embryogenesis. The Smad1 knockout mouse shows impaired allantois formation and also markedly reduced primordial germ cell (PGC) formation [12,120], whereas Smad2 deleted mutant mice have defects in mesoderm induction, left-right patterning and craniofacial development [121,122]. Interestingly, Dunn et al., recently showed that deleting the exon 3 of the full length Smad2 restores its DNA binding ability, and that homozygous mice exclusively expressing the short isoform Smad2(Δ exon3) are viable and fertile [16]. Consequently, the short isoform of Smad2, or the replacement of the coding sequence of full length Smad2 with closely related Smad3, can activate all essential target genes downstream of TGF-β-related ligands [16]. The tumour suppressor Smad4 is required during gastrulation and later in the anterior development of the mouse embryo as shown by the Smad4 knockout mouse [123]. Smad5 knockout mice have multiple embryonic and extra-embryonic defects [124]. Interestingly, homozygous Smad5 knockout mice that die at midgestation also show a greatly reduced number or total loss of PGCs [13], similar to what happens in the BMP4 and -8b knockout mice [3].

Two different Smad3 knockout mice have been generated with disruptions in either the exon 2 or exon 8. In contrast to the other R-Smad knockout mice, the Smad3 deficient mouse is viable displaying various defects, such as accelerated wound healing (KO in exon 8) [125], impaired immune function and diminished responsiveness of T cells to TGF-β (KO in exon 8) [126] and a predisposition to colorectal adenocarcinomas (KO in exon 2) [127]. Zhu et al. reported that the Smad3 null mice with deletion of exon 2 are fertile but produced smaller litters [127]. In contrast, the ovarian function in the Smad3 null mice harbouring a deletion in the exon 8 has been reported to be abnormal. The mice exhibit reduced fertility and circulating estrogen levels due to impaired folliculogenesis, and also underdeveloped mammary glands compared to female wild type mice [62,128]. The primordial pool of follicles at birth is not affected by the mutation and the ovaries appear similar to the wild type mouse but later in postnatal life the ovaries of Smad3 deficient mice have higher numbers of primordial follicles and fewer large preantral and antral follicles than wild type mice, suggesting that the absence of Smad3 may delay follicular maturation [62,126]. More detailed study of the Smad3 knockout mouse ovaries has revealed that Smad3 deficiency slows follicle growth, causes atretic follicles, degenerated oocytes and low expression of the anti-apoptotic protein bcl-2 [128]. In addition, Smad3 deficiency affects follicular differentiation as indicated by decreased expression of estrogen receptor β and inhibin α subunit as well as increased expression of estrogen receptor α [128]. Estradiol levels are low whereas FSH levels are high. The reason for this discrepancy between the two Smad3 null mouse lines remains unknown. The knockout mouse for the inhibitory Smad, Smad6, is also viable but has cardiovascular abnormalities, suggesting a role for Smad6 in the development and homeostasis of the cardiovascular system [129]. Smad7 or Smad8 knockout mice have not been reported as of yet.

Bristol-Gould et al. recently reported the introduction of a dominant negative Smad2 gene into mouse gonads [130]. Expression of the transgene under the AMH promotor directs the expression of this gene to the granulosa cells and causes subfertility, decreased litter sizes and breeding frequency in the mutant female mice. The transgenic ovaries contain fewer corpora lutea compared to ovaries from normal littermates and develop multioocytic follicles. In addition, the transgenic ovaries exhibit symptoms of premature aging and develop multiple small lesions or inclusion cysts in only three months. The Smad2 dominant negative transgenic mouse bears resemblance to the inhibin α-subunit transgenic mouse in being subfertile, as well as in developing cysts and multioocytic follicles [131]. The ovarian phenotype of these mice resembles a human condition known as endosalpingiosis, a pelvic condition typified by the presence of cystic glandular structures lined by benign tubal/salpingeal epithelium supporting a TGF-β/activin/Smad2-dependence in the onset of this disease [130]. Taken together, it is known that Smad3 plays a role in folliculogenesis but the role of other Smads is not so clear since many of the Smad knockout mouse models die *in utero *during early embryonic development. Generation of tissue-specific conditional and/or inducible knockout models or the use of RNAi techniques might be useful in determining the specific importance of each of the Smads at different stages of folliculogenesis.

## Conclusion

Several TGF-β superfamily ligands play important roles in ovarian organogenesis and the process of folliculogenesis. Especially the BMPs are prominent during ovarian organogenesis, whereas folliculogenesis seems to be regulated by a complex interplay between different GDFs, BMPs and other TGF-β-like ligands. Some of these ligands are expressed only within the ovary, such as GDF-9B and AMH, and also some of their receptors are ovary-specific, e.g. AMHRII, but most ovarian expressed growth factors have diverse roles in other tissues as well. Smads, however, are ubiquitously expressed in nearly all cell types in the body. Therefore the ovary-specific Smad-interacting proteins, such as transcription factors and co-modulators, may play a prominent role in the TGF-β superfamily ligand target gene selection in the ovary. The family of Smad proteins was discovered 10 years ago but we are still in the early days of understanding Smad function in the ovary and in fertility. There is the possibility for research into the development of small molecule drugs against the Smads and their interacting partners, and it would be interesting to see in the future if these molecules would affect the different stages of follicle development and whether they could be used to treat infertility.

*Note added in proof*: A very recent paper by Massague et al. provides an excellent review on the role of Smads as transcription factors (Massague et al., **Smad transcription factors. ***Genes Dev *2005, **19**:2783–810).

## Supplementary Material

Additional File 1 GoCore v 5.0.1  summary of an alignment of ninety-one Smad sequences, representing eight Smad proteins across seventeen mammalian species. The species included are H. sapiens, P. troglodytes, P. anubis, P. pygmaeus, M. mulatta, C. familiaris, S. scrofa, B. taurus, O. aries, E. caballus, L. africana, R. norvegicus, M. musculus, E. telfairi, D. novemcinctus, M. vison, and O. cuniculus. Where sequence variants exist, the longest variants are included. For clarity, the summary is superimposed upon the human sequences. Dark grey shading represents a region of 120 residues in the alignment that only exist in the inhibitory Smads and is not displayed. Light grey residues in Smad2 represent the exon 3 insertion splice variant. Other shading represents residues that are found uniquely conserved in particular groups of Smads across at least 80% of the tested species. Unique, conserved residues are shaded dark blue for the group of Smads 1, 5 and 8, dark green for Smads 2 and 3, light blue for all receptor-mediated Smads, light green for all non-inhibitory Smads, tan for all inhibitory Smads, orange for all Smads except Smad7, and yellow for all Smads. The MH1 and MH2 domains are boxed in black and labelled accordingly. The L3 loop in the MH2 domain is boxed in blue, and the PY motifs in the linker region are boxed in red. Click here for file
