# Supplementary figures and images for: Smad signalling in the ovary
Source: Reprod Biol Endocrinol. 2006 Apr 12;4:21. doi: 10.1186/1477-7827-4-21 (PMC1459162; doi:10.1186/1477-7827-4-21)

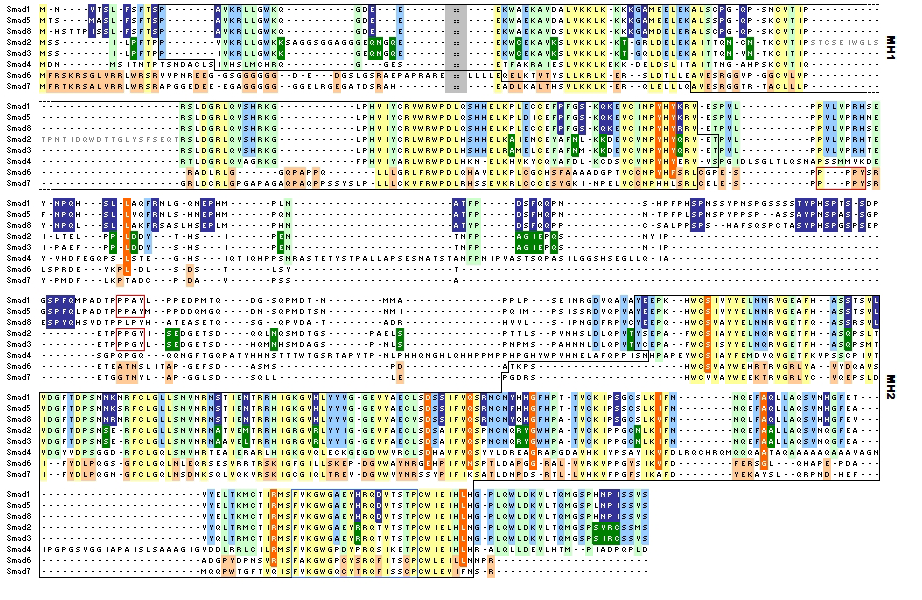

Supplement: Additional File 1 — GoCore v 5.0.1 summary of an alignment of ninety-one Smad sequences, representing eight Smad proteins across seventeen mammalian species. The species included are H. sapiens, P. troglodytes, P. anubis, P. pygmaeus, M. mulatta, C. familiaris, S. scrofa, B. taurus, O. aries, E. caballus, L. africana, R. norvegicus, M. musculus, E. telfairi, D. novemcinctus, M. vison, and O. cuniculus. Where sequence variants exist, the longest variants are included. For clarity, the summary is superimposed upon the human sequences. Dark grey shading represents a region of 120 residues in the alignment that only exist in the inhibitory Smads and is not displayed. Light grey residues in Smad2 represent the exon 3 insertion splice variant. Other shading represents residues that are found uniquely conserved in particular groups of Smads across at least 80% of the tested species. Unique, conserved residues are shaded dark blue for the group of Smads 1, 5 and 8, dark green for Smads 2 and 3, light blue for all receptor-mediated Smads, light green for all non-inhibitory Smads, tan for all inhibitory Smads, orange for all Smads except Smad7, and yellow for all Smads. The MH1 and MH2 domains are boxed in black and labelled accordingly. The L3 loop in the MH2 domain is boxed in blue, and the PY motifs in the linker region are boxed in red. [file 1477-7827-4-21-S1.png]
